# Design and Performance of Property Gradient Ternary Nitride Coating Based on Process Control

**DOI:** 10.3390/ma11050758

**Published:** 2018-05-09

**Authors:** Pei Yan, Kaijie Chen, Yubin Wang, Han Zhou, Zeyu Peng, Li Jiao, Xibin Wang

**Affiliations:** Key Laboratory of Fundamental Science for Advanced Machining, School of Mechanical Engineering, Beijing Institute of Technology, Beijing 100081, China; neuchenkaijie@163.com (K.C.); wangyubin86@126.com (Y.W.); Zhchow1314@163.com (H.Z.); pzypzy12345@163.com (Z.P.); jiaoli@bit.edu.cn (L.J.); cutting0@bit.edu.cn (X.W.)

**Keywords:** property gradient, gradient coating, process control, friction and wear, cutting performance, wear mechanism

## Abstract

Surface coating is an effective approach to improve cutting tool performance, and multiple or gradient coating structures have become a common development strategy. However, composition mutations at the interfaces decrease the performance of multi-layered coatings. The key mitigation technique has been to reduce the interface effect at the boundaries. This study proposes a structure design method for property-component gradient coatings based on process control. The method produces coatings with high internal cohesion and high external hardness, which could reduce the composition and performance mutations at the interface. A ZrTiN property gradient ternary nitride coating was deposited on cemented carbide by multi-arc ion plating with separated Ti and Zr targets. The mechanical properties, friction behaviors, and cutting performances were systematically investigated, compared with a single-layer coating. The results indicated that the gradient coating had better friction and wear performance with lower wear rate and higher resistance to peeling off during sliding friction. The gradient coating had better wear and damage resistance in cutting processes, with lower machined surface roughness Ra. Gradient-structured coatings could effectively inhibit micro crack initiation and growth under alternating force and temperature load. This method could be extended to similar ternary nitride coatings.

## 1. Introduction

With the rapid development of high- and ultra-high-speed cutting and dry cutting technology, and the constant emergence of new engineering materials with excellent mechanical properties, the condition of the cutting deformation zone is becoming extremely harsh [1]. Consequently, better performances are demanded of cutting tools, including hardness, strength, and heat-resistance. As an efficient and economical approach to improving the performances of existing materials [2,3,4], surface coating technologies have been rapidly developed and widely applied in coated tools [5,6,7]. After several decades of development, different coating materials and structures have been invented, and multiple-layer or gradient-layer structures have become the development tendency of coating technology [8,9,10,11].

The cutting area in actual cutting processes must withstand extremely high force and temperature [12], and there are intensive interactions between the tool, workpiece, and coating at two interfaces: tool–coating and workpiece–coating. The coating is subjected to enormous external extrusion and shearing effects at the workpiece–coating interface, and high temperature and thermal shock at the tool–coating interface [13]. Excellent comprehensive mechanical performances of the coatings, such as compressive property, shear resistance, and thermal deformation resistance, are demanded. Theoretically, this requires the highest hardness of the outer layer, and the highest adhesive strength of the inner layer—a so-called hardness–adhesive strength gradient distribution.

However, the substrate and coating are different materials, and it can be considered that the coating is obtained through the surface growth effect. There is usually a boundary (or interface) between different materials with different mechanical performances [14,15,16], and their interaction is usually expressed as “adhesive”. For multi-layer coatings or composite coatings, there are even multiple boundaries. Under the effect of temperature, alternating temperature, humidity, and other external stresses, there may be solid–solid diffusion, ion charge migration, hot electron injection, electrochemical corrosion, and even cracks at the boundary, leading to a change of the interface’s thermal and mechanical properties until failure. For instance, as the materials’ thermal expansion coefficients are not the same, the deformation difference of the materials beside the interface will cause tangential residual stress or even cracks under high cutting temperature, and the fatigue properties of the coating will be worsened.

Because of the interface and the adhesive, crack initiation, propagation, and even coating fall-off often occur at the interface in practical application [17], which have a serious impact on the comprehensive performance of the coating. If the adhesive force is poor, the coating will also be subject to severe peeling off and rapid failure, even if the coating itself has high hardness and toughness [18]. So, one of the key techniques is to improve the bonding strength and reduce the interface effect. In the available literature, the composition and performance of the interface effect can be reduced by adding a metal transition layer (the bonding layer between coating and substrate) or making a reasonable selection of the middle layer [15,19,20]. However, the interface effect of the coating is still difficult to effectively control for multiple-layer or gradient-layer coatings.

In this study, a structure design method for property-component gradient coatings based on process control is proposed, with high internal cohesion and high external hardness. This material system can reduce the composition and performance mutations at the interface. A typical property gradient ternary nitride coating was designed and prepared by multi arc ion plating (MAIP), and the mechanical properties, friction behaviors, and cutting performances were systematically investigated. This method could be extended to similar hard coating materials, such as the ternary nitride coating material Me_1_-Me_2_-N (Me_1_/Me_2_ are two different metal elements such as Ti, Al, Zr, Cr, and so on). The corresponding gradient coatings can be designed and prepared according to different practical applications conditions.

## 2. Design Method and Experimental Procedures

### 2.1. Structure Design Method of the Gradient Coating

The relationship between the basic mechanical properties (in particular, hardness and binding strength) of the coatings and the preparation parameters were obtained based on uniform deposition experiments on the evaluated coating material. Then, the parameters were selected under which both the hardness and binding strength of these coatings were acceptable. The design of gradient structure followed the principle that the coating had the highest hardness in its outer layer and the highest adhesive strength in its inner layer, and proper intermediate coatings could be selected and added. The appropriate deposition process and parameters were selected to ensure that the coating structure had a hardness–adhesive strength gradient distribution, under the precondition of process operability and comprehensive properties of coating. Then, the appropriate parameters of the intermediate layers could be chosen according to the process parameter range of each layer. In order to avoid the mutation of properties at the interfaces, the process parameters on both sides of the interface should provide a moderately uniform transition. Besides, according to the different element contents of the coating and substrate, the metal transition layer was also gradient-designed. The preparation parameters and sequence should be determined on the basis of the structure’s design.

The gradient coatings could be deposited just by adjusting the parameters during the preparation process. Compared with the existing coating design method, this gradient coating was the same material system, with an effective transition at the interface. The element composition, physical and chemical properties, and microstructure of the coating achieved a continuous transition from the surface to the interior, so as to effectively alleviate the stress concentration at the interface. The optimization of bonding strength and surface hardness could effectively improve the comprehensive mechanical properties of the coating, which could effectively decrease the stress concentration caused by mismatch, reduce the coating failure, and improve the reliability of the coating under application. Besides, the material system was simple, with low equipment and raw material requirements.

### 2.2. Design and Preparation of Gradient ZrTiN Coatings

**Figure 1:**
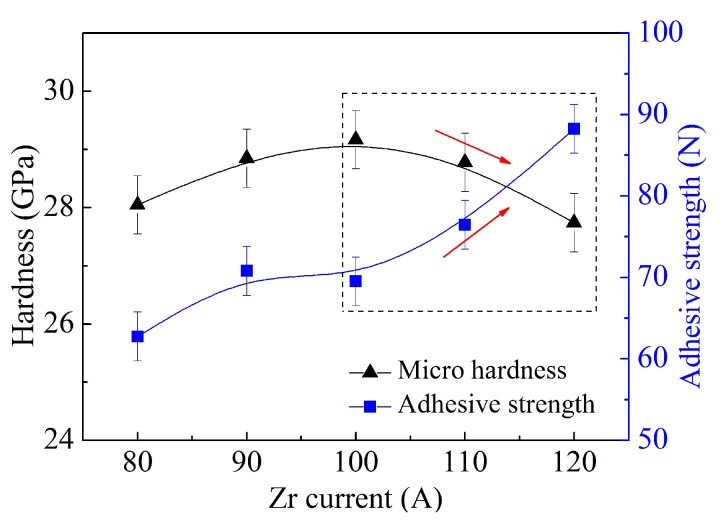
The hardness and adhesive strength of the coatings varied with Zr current [23].

The ternary nitride coating selected was ZrTiN, a stable substitutional solid solution with even better mechanical properties than AlTiN [17,21,22]. The substrate employed was cemented carbide YT15 (WC + 15%TiC + 6%Co). Two kinds of samples were employed in this research: the disk-shape specimen with size of Ø 56 × 4 mm, and square insert with size of 16 × 16 × 4 mm. A multifunctional coating equipment was employed to deposit the ZrTiN coatings by multi-arc ion plating method, with two separated controlled Ti and Zr targets (purity 99.9%). The reactive gas selected was high-purity nitrogen (99.999%), and the working gas which sputtered the metal target or aided in arc ignition was high-purity argon (99.999%). During the deposition process, the deposition chamber was kept at a pressure of about 0.45 Pa, with a mass flow meter to control the gas flows. Before the coating process, the coating chamber was vacuumed to a pressure lower than 1.0 × 10^−3^ Pa and progressively warmed up to the predetermined temperature at the same time. Then, the substrate surface was cleaned by ion bombardment for about 2 min with bias voltage −800 V. The metal transition layer and the coating were deposited, and the target current, nitrogen flow, depositing time, and negative substrate bias could be adjusted as needed. Combining existing completed process experimental results investigated previously [23], the optimized deposition parameters for single layer coating were: N_2_ flowrate 120 sccm, Zr target current 110 A, Ti target current 70 A, bias voltage −150 V, temperature 200 °C, and deposition time 75 min. When the other parameters were fixed and the sum of the targets was 180 A, the property curves of the coatings varied with Zr current as denoted in Figure 1 [23]. The grain size of the coatings was around 10 nm, estimated by Debye–Scherrer formula according to X-ray diffraction (XRD, D8 ADVANCE, Bruker, German) test.

The gradient design follows the principle that the bonding strength of the most inner layer should be the highest, and the hardness of the outer layer should be the highest. It was obvious that when the Zr current increased from 100 A to 120 A, the adhesive strength increased and the hardness decreased gradually (shown as red arrows on the hardness and adhesive strength curves in Figure 1), which could meet the requirements of hardness–adhesive strength gradient coating. A typical three-layer coating structure was selected, and the parameter range selected is denoted in the dotted box. The Zr target current of the outermost coating was 100 A, and that of the inner layer was 120 A, with middle layer Zr current 110 A. From the innermost layer to the outermost layer of this coating structure, the adhesive strength, hardness, and composition difference gradient changed without obvious mutation, and gradually decreased by controlling the Zr current. To further reduce the discontinuity at the interface, the deposition parameters were fine-adjusted at two minutes before and after each layer of the coating.

The gradient structure was also applied for the metal transition layer. Ti was selected as the innermost layer of the metal transition layer, which could form a semicoherent interface with Ti element in the substrate, and a compound interface with C element in the substrate. The outermost layer of the transition layer was Ti/Zr alloy, with the same target current of the inner layer of the ZrTiN coating. The deposition parameters of the intermediate metal layer were also uniformly changed. According to the design of the gradient coating and the optimization of the interface, the final deposition process parameters were obtained as shown in Table 1. The specific parameters of the deposition process were adjusted according to this. This gradient coating was labeled as ZGC. A single layer of ZrTiN coating (SZT) without transition or gradient layer was also deposited as the reference group.

The morphologies of the surfaces and cross sections of the deposited coatings were analyzed with a scanning electron microscope (SEM). The elements distribution and crystal structure were obtained from EDX and XRD. Binding strength (or adhesive strength) and thickness of the coating were measured using a scratch test on an MFT-3000 surface properties tester, and tests were repeated four times and averaged. The microhardness was obtained on a MH-6 hardness tester with the load of 0.25 N on three different specimens and four points per sample.

### 2.3. Friction Test

Friction and wear properties were evaluated on a high-speed nano-micro tribometer (UMT-2, CETR), using ball-on-disk method at room temperature. Each experiment was repeated two times, without lubricant used during friction. The polished ball (Ø 9.525 mm) was 40Cr (GB/T 3077-1999) hardened steel (HRC ~55) with a surface roughness Ra 0.05 μm. The ball and disk were both ultrasonically cleaned in alcohol and then acetone before the experiments. The ball was fixed and the disk was rotated to ensure that the sliding speeds at the friction contact zone were between 40–140 m/min, and the load was among 5–25 N in the tests. The friction coefficients could be obtained by the tribometer. The wear rate *W* was defined as *W* = *V*/(*PL*) (mm^3^/Nm), where *V* was the loss of volume, *P* was the load, and *L* was the friction distance. The worn patterns of the coating were investigated by SEM and Optical Profiler (Wyko NT9300).

### 2.4. Cutting Test

The single factor cutting experiments were carried out on a traditional CA6140 lathe without cutting fluid, and each experiment was repeated twice. The workpiece material was 40Cr hardened steel (HRC 45~50), with the dimension of Ø 100 × 400 mm. The cutting parameter ranges were cutting speed 60–200 m/min, feed rate 0.10–0.30 mm/rev, depth of cut 0.15–0.35 mm, cutting distance 300 m. The cutting tools were EGC and TGC coated cemented carbide inserts with the same geometry: rake angle *γ*_o_ 15°, clearance angle *α*_o_ 5°, inclination angle *λ*_s_ 2°, entering angle *K*_r_ 45°, cutting edge radius *γ*_ε_ 0.5 mm. Cutting forces were obtained by a Kistler 9265A piezoelectric quartz dynamometer. Each cutting force was measured three times and averaged. Roughness Ra was measured by a Times TR200 roughness tester at four positions symmetrically distributed on the specimen, then the average was taken. Cutting temperature was measured ten times each experiment by a thermal infrared imager (NEC TH5104R), then the average was taken. Wear areas of the tool were measured by a microscope system. The worn areas of the tools were investigated by SEM and EDX.

## 3. Results and Discussion

### 3.1. Structure and Property of the Coatings

**Figure 2:**
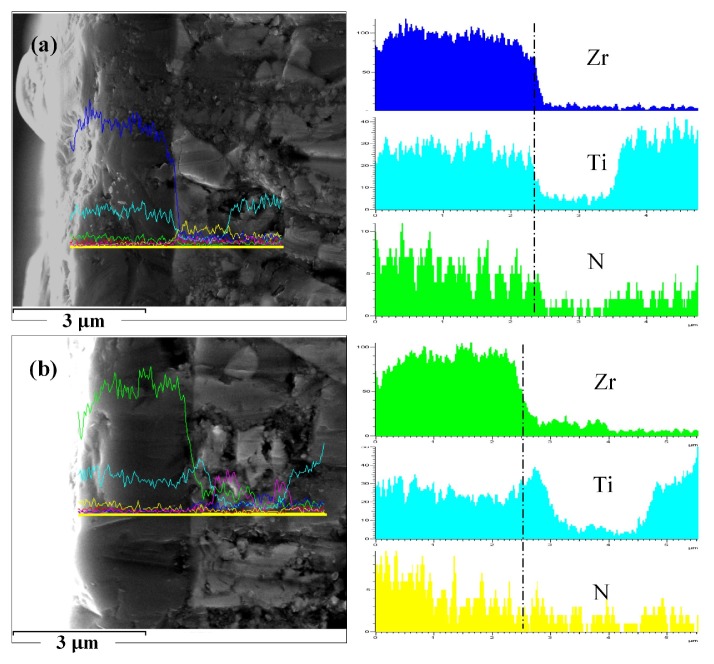
The SEM micrographs of sample cross section and EDX line scanning analysis results of Zr, Ti, and N elements of the coatings: (**a**) SZT (single-layer ZrTiN coating); (**b**) ZGC (property-component gradient ZrTiN coating).

**Figure 3:**
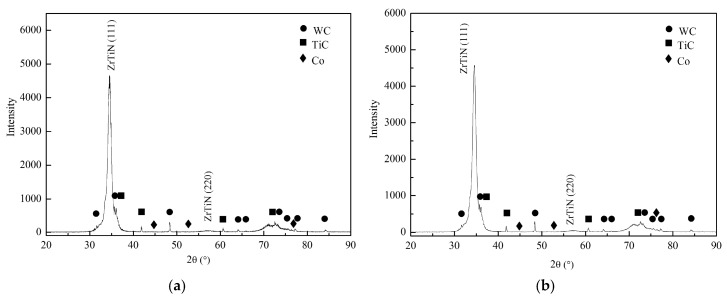
X-ray diffraction analysis of the coatings: (**a**) SZT; (**b**) ZGC.

Figure 2 shows the cross section micrographs of these coatings and EDX line scanning analysis results. Both coatings showed dense and fine structures without common columnar crystals, which benefited from the solid solution mechanism. Besides, the coatings were bonded to the substrate very well. As designed, from the outside to the inside in ZGC coating, the content of Zr increased gradually and that of Ti decreased; the Ti content at the interface (shown as the dotted line) was obvious, and it decreased in a small area outside the interface with increasing Zr content. Results showed a gradient distribution of the elements, almost in agreement with the structure design and depositing process. As a single-layer coating, the elements in the SZT coating were uniformly distributed in the thickness direction of the coating and the transition layer. Figure 3 displays the XRD patterns of the coatings. They indicate that only face centered cubic phase of ZrTiN existed, with preferentially crystal direction (111) along the direction perpendicular to the substrate surface. The crystal structure of the coatings was basically the same.

Mechanical properties of the coatings such as micro hardness, binding strength, and thickness are listed in Table 2. The hardness of both coatings was over 31 GPa, which is much higher than that of TiN or ZrN (about 21–23 GPa). The adhesive strength of ZGC showed an increase trend compared with SZT, which was because of the selected depositing parameter of the inner layer as well as the metal transition layer.

### 3.2. Friction and Wear Behavior of the Coatings

**Figure 4:**
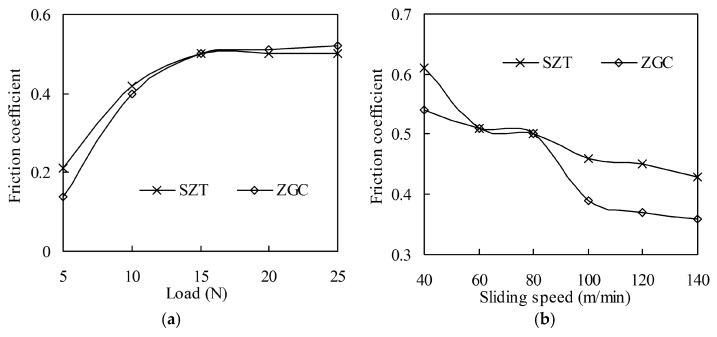
Friction coefficient of the coatings under different loads (speed 80 m/min) and sliding speeds (load 15 N). (Test time 5 min): (**a**) Friction coefficient under different loads; (**b**) Friction coefficient under different sliding speeds.

The friction and wear behaviors of these coatings under different applied load (5, 10, 15, 20, 25 N) and sliding speed (40, 60, 80, 100, 120, 140 m/min) were studied systematically. According to friction coefficient curves during the experiments, the friction process of SZT against 40Cr hardened steel were not smooth, with an obviously fluctuating friction coefficient, especially at low load or low speed. The variation of the average friction coefficients with load and sliding speed are shown in Figure 4. In general, the friction coefficient increased with the increase of applied load, while it decreased with friction speed. The two coatings showed little difference at different loads, but ZGC showed a lower friction coefficient at high speed (over 80 m/min).

**Figure 5:**
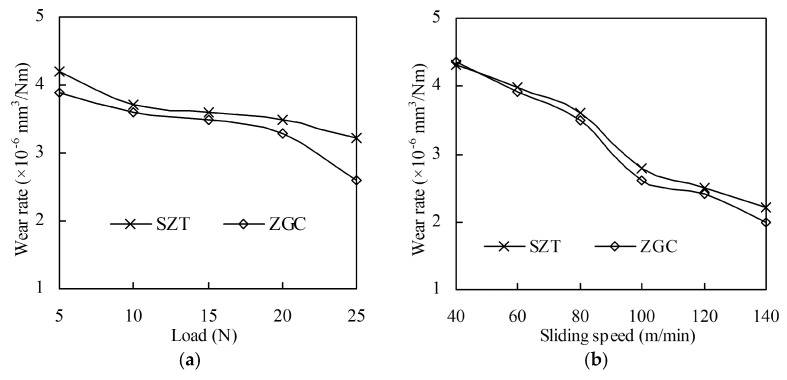
Wear rate of the coatings under different loads (speed 80 m/min) and sliding speeds (load 15 N): (**a**) Wear rate under different loads; (**b**) Wear rate under different sliding speeds.

Figure 5 illustrates that the wear rate of these coatings varied with different applied load and sliding speed. The figure shows that the wear rate decreased with both increased load and sliding speed. This is because the friction force and temperature increased rapidly as the load and friction speed increased, resulting in severe wear and surface softening of the 40Cr ball in the contact zone. The actual contact area increased, which brought down the pressure of the actual contact zone and reduced the wear of the coated disk. ZGC showed a lower wear rate under all experimental conditions, benefiting from its gradient structure and better adhesive strength.

**Figure 6:**
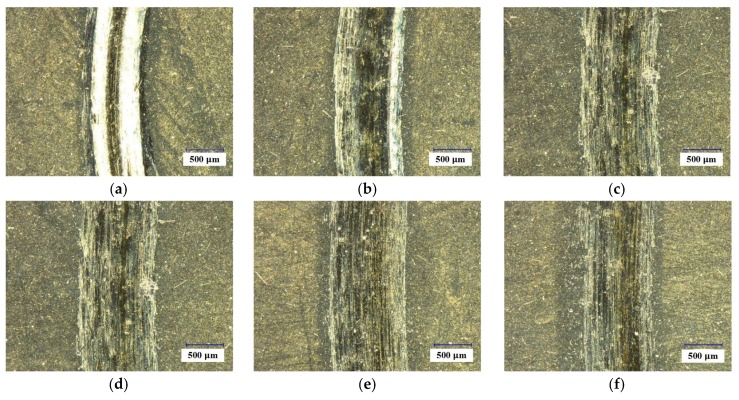
Micrographs of the wear surfaces of ZGC under different speeds. (**a**) 40 m/min; (**b**) 60 m/min; (**c**) 80 m/min; (**d**) 100 m/min; (**e**) 120 m/min; (**f**) 140 m/min. (Load 15 N, sliding time 5 min).

**Figure 7:**
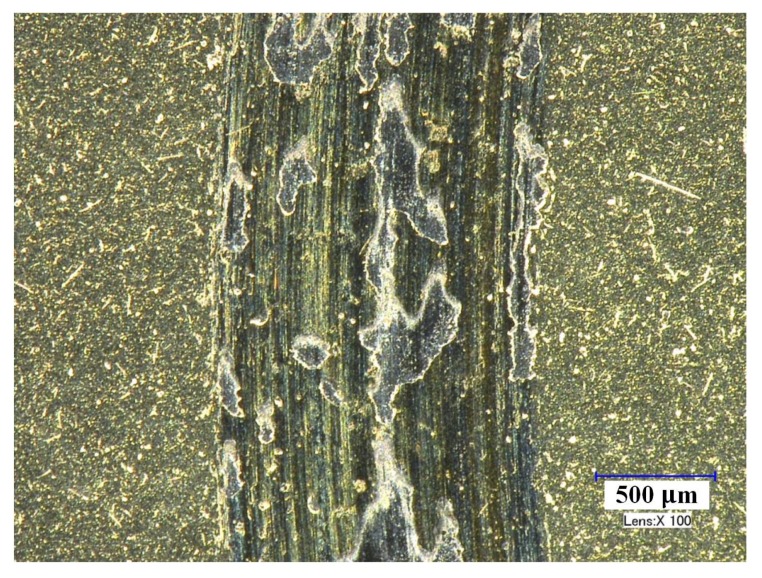
The SZT coating breaking off at the wear track (speed 60 m/min, load 10 N, sliding time 5 min).

Figure 6 shows micrographs of the worn surfaces of ZGC under different speeds. The wear tracks of the coating were all smooth without adhered material or broken-off coating. As the friction speed increased, the wear track became even slighter, but the width of the wear scar increased at the same time. Under these experimental conditions (especially high speed and light load), the tribological properties of ZGC were stable, with very slight wear. In contrast, if the cutting speed was low or the load was high, there might be some breaking-off of the SZT coating, and a typical area is depicted in Figure 7. In general, compared with SZT, the ZGC coating had a better friction performance with lower wear rate and higher resistance to peeling off.

### 3.3. Cutting Performance of the Coatings

**Figure 8:**
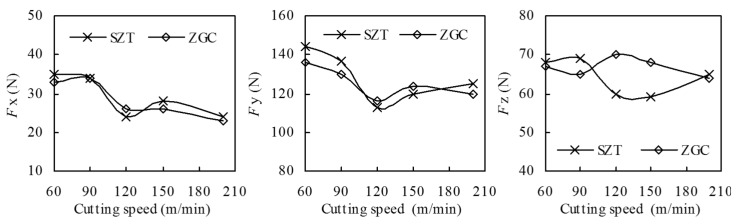
Effect of cutting speed on cutting forces of the ZrTiN-coated inserts (*a_p_* = 0.15 mm, *f* = 0.10 mm/rev, cutting distance *L* = 300 m).

The effects of different coated tools on cutting forces and temperatures were systematically investigated according to single-factor experiments. Figure 8 indicates that the three-dimensional cutting forces *Fx*, *Fy*, and *Fz* varied with cutting speeds during the machining of 40Cr hardened steel (*a_p_* = 0.15 mm, *f* = 0.10 mm/rev). It was obvious that cutting forces of the coated inserts were decreased with increased cutting speed, which complied with the metal cutting principle. Because the actual cutting tests were only carried out over a short distance, the tool wear was slight; together with small differences of the coatings’ friction coefficients, the cutting force difference between these two coatings in the cutting process was not obvious. The experiments with varying feed rate and cutting depth showed again that the cutting force of the two coatings was almost the same.

**Figure 9:**
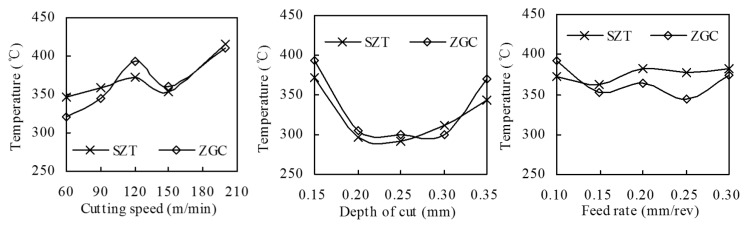
The highest cutting temperature at the tool–chip interface of ZrTiN-coated inserts at different cutting conditions.

Figure 9 shows the averaged highest cutting temperature measured at the cutting zone of different tools under different cutting conditions. Following the metal cutting principle, the averaged cutting temperature increased with increasing cutting speeds, cutting depth, and feed rate. The influence of the two coatings on cutting temperature was not significant.

**Figure 10:**
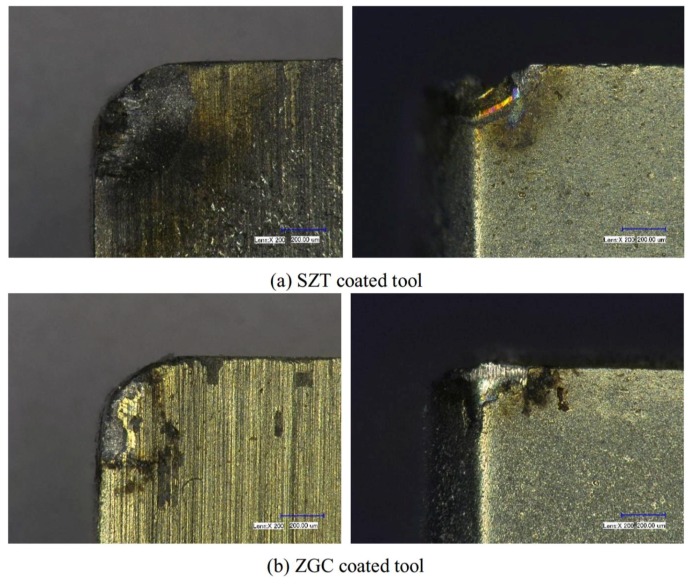
Wear morphology of rake face and flank face at a cutting speed of 200 m/min.

However, wear and damage resistance of the two coatings under extreme cutting conditions were significantly differentiated. Figure 10 indicates the wear of the rake and flank faces of the coated tool under the cutting speed of 200 m/min. As shown in Figure 10a, it was obvious that the damage and peeling off of SZT was serious—even the tool nose was broken. Besides, there were a large amount of chip sticking onto the rake face. The wear and peeling off of the ZGC coating was slighter, with the tool nose of the substrate basically staying in its original condition. As indicated in Figure 10b, ZGC showed a better performance than SZT under the condition of high-speed cutting due to the gradient structure and high adhesive strength, which could reduce the thermal deformation and thermal damage under high cutting temperature.

**Figure 11:**
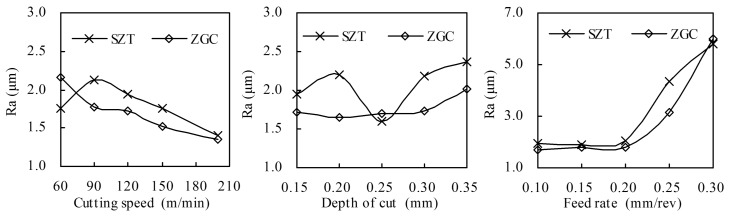
Ra of the machined surface at different cutting conditions (cutting distance *L* = 300 m).

Figure 11 illustrates the averaged Ra of the machined surface of the coated tools under different cutting parameters. Plainly, Ra decreased with the increase of cutting speed, and increased with the increase of depth of cut and feed rate. The feed rate had the most significant impact on the roughness. Compared with SZT, ZGC gradient-coated inserts could reduce surface roughness Ra. This is because the adhesive strength of ZGC was higher than SZT, and the gradient structure could effectively resist the thermal wear and damage during the cutting process. Besides, the material bonding of ZGC was slight, which could also improve the surface quality.

**Figure 12:**
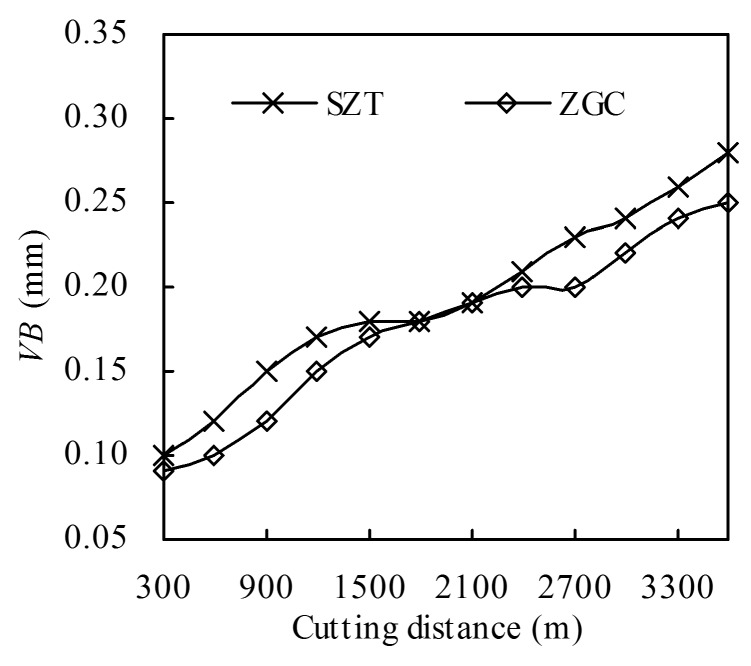
Flank wear *VB* of the coated tools in dry machining of 40Cr hardened steel (*v* = 120 m/min, *a*_p_ = 0.20 mm, *f* = 0.10 mm/rev).

Figure 12 indicates the variation in the flank wear of the tools during the machining process until cutting distance *L* = 3600 m under cutting parameters of *v* = 120 m/min, *a_p_* = 0.20 mm, *f* = 0.10 mm/rev. The figure shows that the ZGC coated inserts had a better performance in flank wear resistance than SZT, which corresponded to higher adhesive strength and gradient structures. The gradient structure of the ZGC coatings could effectively inhibit the micro crack initiation and growth under alternating force and temperature load.

**Figure 13:**
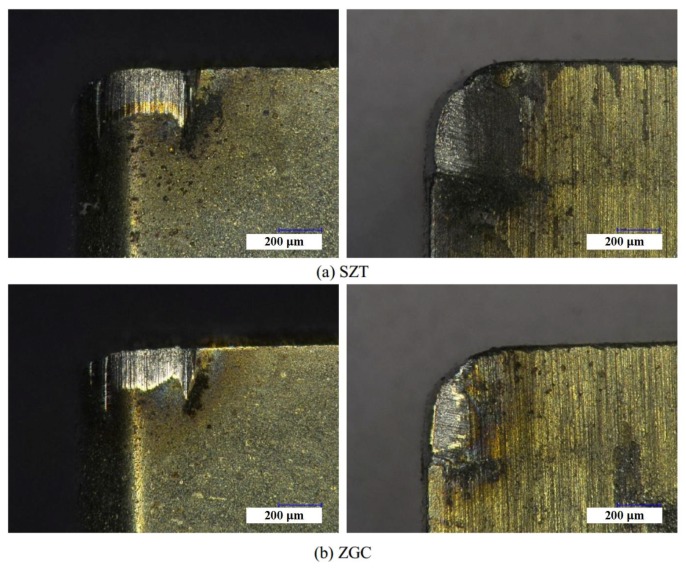
Micrographs of the worn flank face and rake face (*v* = 120 m/min, *a*_p_ = 0.20 mm, *f* = 0.10 mm/r, *L* = 3600 m).

**Figure 14:**
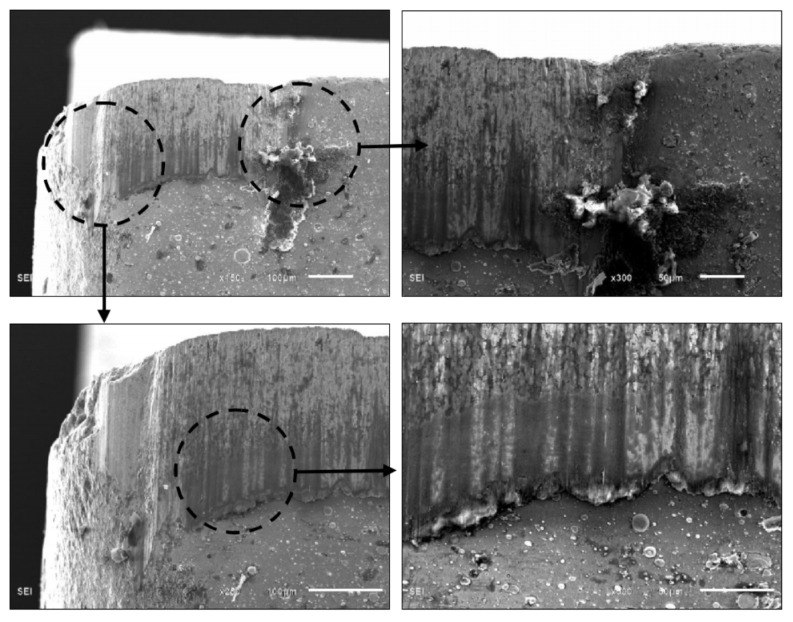
Micrographs of worn flank face of SZT (*v* = 120 m/min, *a*_p_ = 0.20 mm, *f* = 0.10 mm/r, *L* = 3600 m).

Figure 13 shows micrographs of the worn areas of the flank and rake faces of the coated tools. It is obvious that the main wear mechanisms of the flank face were abrasive wear and boundary wear. The flank wear of SZT was more serious, with workpiece materials obviously bonded at the wear area away from the cutting edge, as demonstrated in Figure 14. The wear of the flank face was mainly due to the pressure and friction between the machined surface and the tool. The hardness of the ZGC surface was higher with better adhesive strength, which meant better friction resistance, so the flank wear was slight. Although the hardness of SZT was better, the bonding strength was low with severe micro collapse and spalling, resulting in greater flank wear.

**Figure 15:**
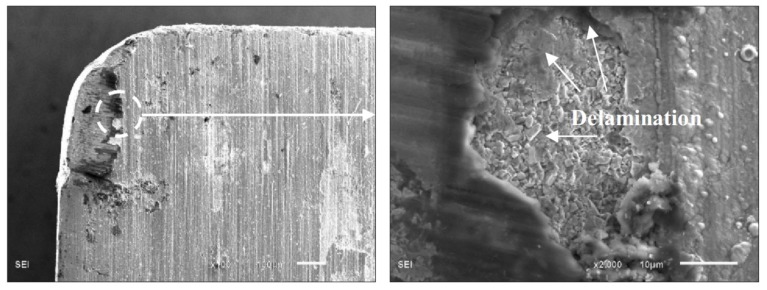
Delamination micrograph of ZGC coating on the rake face.

As shown in Figure 13, the main wear mechanism of the rake face was crater wear and adhesive abrasion. In the cutting process, a great deal of friction heat was produced by the severe friction effect between the chip and rake face, resulting in high temperature near the crater with a large temperature gradient and thermal shock. There was also great thermal stress in the coatings. Due to the lack of a metal transition layer, the coating on the rake face near the crater flaked seriously under the repeated extrusion and friction of chips. For ZGC coatings, there was some slight delamination in the chip flow direction of the crater edge, as shown in Figure 15. Delamination of the coating could effectively avoid flaking, and the non-peeling part of the coating could still protect the tool effectively. So, the ZGC coating had a better crater wear resistance, and the crater wear area was relatively small.

## 4. Conclusions

A structure design method of a property-component gradient coating based on process control was proposed, a typical ZrTiN coating was designed and prepared, and the mechanical properties, friction behaviors, and cutting performance were systematically investigated. The main conclusions are as follows:

(1) The property-component gradient which was designed and prepared by adjusting parameters during the deposition process could effectively improve the performances of the coatings.
(2) The gradient coating ZGC had a better friction and wear performance with a lower wear rate and higher resistance to peeling off during sliding friction.
(3) The gradient coating ZGC had a better wear and damage resistance in the cutting process, with lower machined surface roughness Ra.
(4) A gradient structure in the coatings can effectively inhibit micro crack initiation and growth under alternating force and temperature load, with some slight delamination.

## Figures and Tables

**Table 1:** Preparation process and parameters of ZrTiN gradient coating.

Step.	Layer or Interface	Ti Current (A)	Zr Current (A)	N_2_ Flowrate (sccm)	Time (min)
1	Innermost transition	60	0	0	2
2	Intermediate transition	60	90	0	2
3	Outermost transition	60	120	0	2
4	-	60	120	60	2
5	Innermost coating	60	120	120	25
6	-	64	116	120	2
7	-	67	113	120	2
8	Intermediate coating	70	110	120	25
9	-	74	106	120	2
10	-	77	103	120	2
11	Outermost coating	80	100	120	25

**Table 2:** Mechanical properties of the coatings.

Coating	Micro Hardness (GPa)	Adhesive Strength (N)	Thickness (μm)
SZT	32.6	76.4	2.70
ZGC	31.7	83.2	2.67
